# Invasive characteristics of human prostatic epithelial cells: understanding the metastatic process

**DOI:** 10.1038/sj.bjc.6602325

**Published:** 2005-01-25

**Authors:** C A Hart, M Brown, S Bagley, M Sharrard, N W Clarke

**Affiliations:** 1 PromPT Genito-Urinary Cancer Research, Cancer Research UK Paterson Institute for Cancer Research, Christie Hospital NHS Trust, Wilmslow Road, Manchester M20 4BX, UK; 2 Advanced Imaging Facility, Cancer Research UK, Paterson Institute for Cancer Research, Christie Hospital NHS Trust, Wilmslow Road, Manchester M20 4BX, UK; 3 YCR Cancer Research Unit, Biology Department, The University of York, York YO10 5YW, UK; 4 Department of Urology, Salford Royal Hospital, Eccles Old Road, Salford, UK; 5 Christie Hospital NHS Trust, Wilmslow Road, Manchester, UK

**Keywords:** prostate, metastasis, CXCR4, bone marrow, endothelium

## Abstract

Prostate cancer has a predilection to metastasise to the bone marrow stroma (BMS) by an as yet uncharacterised mechanism. We have defined a series of coculture models of invasion, which simulate the blood/BMS boundary and allow the elucidation of the signalling and mechanics of trans-endothelial migration within the complex bone marrow environment. Confocal microscopy shows that prostate epithelial cells bind specifically to bone marrow endothelial-to-endothelial cell junctions and initiate endothelial cell retraction. Trans-endothelial migration proceeds via an epithelial cell pseudopodial process, with complete epithelial migration occurring after 232±43 min. Stromal-derived factor-1 (SDF-1)/CXCR4 signalling induced PC-3 to invade across a basement membrane although the level of invasion was 3.5-fold less than invasion towards BMS (*P*=0.0007) or bone marrow endothelial cells (*P*=0.004). Maximal SDF-1 signalling of invasion was completely inhibited by 10 *μ*M of the SDF-1 inhibitor T140. However, 10 *μ*M T140 only reduced invasion towards BMS and bone marrow endothelial cells by 59% (*P*=0.001) and 29% (*P*=0.011), respectively. This study highlights the need to examine the potential roles of signalling molecules and/or inhibitors, not just in single-cell models but in coculture models that mimic the complex environment of the bone marrow.

Prostate cancer (CaP) is a widely prevalent disease (Thompson *et al*, 2004) but not all men go on to develop metastases. To do this, it is essential for tumour cells to migrate within the blood and lymphatic system. This is known to occur in other cancers (Taubert *et al*, 2004) and in many men with malignancy in the prostate and in other genito-urinary cancers (McIntyre *et al*, 2000; Meye *et al*, 2002). However, the presence of circulating cells *per se* does not necessarily lead to metastasis formation (Hood and Cheresh, 2002). The reasons for this are unclear at the present time. There is an urgent imperative to gain a better understanding of this process for two simple reasons. Firstly, men who develop bone metastases from CaP will almost invariably die from their disease in the absence of an intercurrent illness. Secondly, there are large numbers of men with a diagnosis of CaP whose disease will remain localised for long periods of time but who are currently being treated aggressively, with inevitable and perhaps unnecessary comorbidity.

The mechanism of metastasis is a complex multistage process that is only beginning to be understood. Initial steps include the loss of cell-to-cell adhesion within the tumour by downregulation of molecular binding complexes such as the E-cadherin/*β*-catenin complex (Umbas *et al*, 1997; Bryden *et al*, 2002) and intravasation of tumour cells through the basement membrane by production of enzymes such as matrix metalloproteinases (Hart *et al*, 2002). Once in the peripheral blood, the circulating tumour cell has to bind at its preferred metastatic site and invade through the local endothelial barrier to gain access to the underlying stroma (Scott *et al*, 2001) where it can then become established (Lang *et al*, 1997, 1998). We have shown previously that prostate epithelial cells preferentially bind to bone marrow endothelial cells in an integrin *β*1-dependent manner, and that only malignant prostate epithelial cells invade in response to bone marrow endothelial cells (Scott *et al*, 2001). However, the specific mechanisms of invasion through the bone marrow endothelial barrier and the stimuli for that invasion are as yet undefined. There is therefore a need for better understanding of this process using *in vitro* models, to allow the identification of the stages and individual components underpinning the metastatic process. Such *in vitro* models would also provide invaluable preclinical tools for the evaluation of new anticancer therapies.

Recent studies have shown that many epithelial cancers metastasise preferentially to the bony skeleton. These include cancers of the prostate (Taichman *et al*, 2002), kidney (Schrader *et al*, 2002), lung (Burger *et al*, 2003) breast (Muller *et al*, 2001) and skin (Robledo *et al*, 2001; Murakami *et al*, 2002). Cells from these tumour types share many of the trafficking characteristics of haematopoietic stem cells (HSC) (Muller *et al*, 2001). The homing of the HSC to the bone marrow during foetal life and after bone marrow transplantation has been well characterised. The key molecular axis for this homing has been shown to involve the CXC chemokine stromal-derived factor-1 (SDF-1 or CXCL12) and its receptor CXCR4 (CD186). This model is supported by the facts that both bone marrow endothelial cells and osteoblasts express SDF-1 (Aiuti *et al*, 1997; Hamada *et al*, 1998; Ponomaryov *et al*, 2000), CXCR4 knockouts do not show haematopoietic engraftment of the bone marrow (Aiuti *et al*, 1999) and that the level of CXCR4 expression by HSC determines their ability to engraft the bone marrow (Peled *et al*, 1999). It has been shown recently that the CXCR4/CXCL12 axis also plays a crucial role in the targeting of several solid tumour metastases, including breast (Muller *et al*, 2001) kidney (Staller *et al*, 2003), lung (Burger *et al*, 2003), pancreas (Koshiba *et al*, 2000) and CaP (Taichman *et al*, 2002; Sun *et al*, 2003) to the bone marrow. It has been shown *in vitro* that CXCR4 and CXCL12 interactions alongside CCR7/CCL21 interactions trigger pseudopodial invasion by malignant breast epithelial cells by actin polymerisation (Muller *et al*, 2001).

The CXCR4/CXCL12 axis is therefore a potential target for therapeutic intervention in malignancies that metastasise specifically to the bone marrow. Neutralisation of CXCR4 with monoclonal antibodies in non-Hodgkin's lymphoma models has been proven to be effective in preventing pseudopodial formation and trans-endothelial migration *in vitro* and to protect against tumour challenge *in vivo*, reducing existing tumour growth while preventing tumour extravasation (Bertolini *et al*, 2002). In this study, we have adapted existing bone marrow invasion models to represent the blood/BMS (BMS) barrier more closely and we have used these to follow malignant prostate epithelial invasion of the bone marrow compartment. Using these models, we have also evaluated the ability of the small peptide inhibitor of CXCR4, T140 (Tamamura *et al*, 2001) to inhibit prostatic invasion *in vitro*.

## MATERIAL AND METHODS

### Materials

All general reagents were purchased from Sigma-Aldridge, Poole, UK. Tissue culture medium and horse serum was from Invitrogen, Paisley, UK with the exception of Ham's F12 media, PAA Laboratories, Austria and EBM-2 Bullet kit from Cambrex Bio Science Ltd, Berkshire, UK. Foetal calf serum (FCS) was supplied by Labtech International Ltd., Uckfield, East Sussex, UK. Matrigel® Basement Membrane Matrix and 8 *μ*M cell culture inserts were from Becton Dickinson Labware, NJ, USA and Worthington trypsin and collagenase type I from Lorne Laboratories Ltd, Twyford, UK. T140 anti CXCR4 peptide was a kind gift from Professor Nobutaka Fujii, Graduate School of Pharmaceutical Sciences, Kyoto University, Sakyo-ku, Kyoto, Japan.

### Antibodies

Mouse anti-human pan cytokeratin was from Sigma-Aldridge, Poole, UK; rabbit anti-mouse biotinylated antibody from DAKO Ltd,. Cambridge, UK and Vectastain Elite ABC kit from Vector Laboratories, CA, USA. CXCR4 clone 12G5 pure and PE conjugate and control IgG2a PE were from BD Biosciences Pharmingen, Oxford, UK.

### Cell lines

The prostate cell lines PC-3 (Kaighn *et al*, 1979) and PNT2-C2 (Berthon *et al*, 1995) were cultured in Ham's F12, 7% FCS and 2 mM L-glutamine and in RPMI 1640, 10% FCS and 2 mM L-glutamine, respectively. PC3-GFP were cultured as standard PC-3 cells but with the addition of Hygromycin B (0.15 mg ml^−1^) (Sharrard and Maitland, 2000). Cultures were grown at 37°C in a humidified atmosphere of 5% CO_2_ in air.

The bone marrow endothelial cell line (BMEC) (Almeida-Porada and Ascensao, 1996) was a gift from Dr Gracia Almeida-Porada (University of Nevada, Reno NV, USA). Bone marrow endothelial cell lines were cultured in EBM-2 Bullet kit/15% FCS and 2 mM L-glutamine (EGM-2). Bone marrow endothelial cell line flasks were precoated prior to cell culture by incubation at 37°C for 1 h with 50 *μ*g ml^−1^ of fibronectin in PBS. Cultures were grown at 37°C in a humidified atmosphere of 5% CO_2_ in air and used up to passage 20. All cell lines were removed from tissue culture flasks by treatment with Trypsin–EDTA.

### Long-term human bone marrow

Bone marrow stroma was cultured from female human ribs removed during routine surgery, after informed consent, for nonmalignant renal disease. Preparation for tissue culture used the method of Coutinho *et al*, 1993. Briefly, bone marrow cells were flushed from the rib, resuspended in long-term culture medium (Iscove's modified Dulbecco's medium, 10% FCS, 10% horse serum and 5 × 10^−7^ M hydrocortisone) before 2 × 10^7^ cells were plated into 25 cm^2^ tissue culture flasks. The cultures were grown at 33°C in 5% CO_2_ in air for 4–5 weeks until haemopoietically active areas were observed. All cells were removed from tissue culture flasks by treatment with trypsin–EDTA.

### Primary prostate epithelial cell cultures

With informed consent prostatic tissue was obtained from male subjects undergoing trans-urethral resection for bladder outflow obstruction arising from CaP or benign prostatic hyperplasia (BPH). Each individual prostate chip was bisected for histological diagnostic evaluation and for tissue culture. Prostate epithelial cells and fibroblasts were isolated by collagenase digestion followed by differential centrifugation (Lang *et al*, 1998). Epithelial cells were grown in flasks in keratinocyte-SFM at 37°C in a humidified atmosphere of 5% CO_2_ in air and then used at passage 1–3. All cells were removed from tissue culture flasks by treatment with trypsin–EDTA.

### Measurement of invasion through BMEC using confocal microscopy

This was carried out using time lapse confocal microscopy measurements. Bone marrow endothelial cell line cells were grown on autoclaved glass coverslips (40 mm) precoated with fibronectin (50 *μ*g ml^−1^) until confluent. The coverslip was then washed and mounted in a Bioptechs FC2 heated chamber closed to the external environment containing EGM-2 media. In all, 5 × 10^5^ PC3-GFP cells in EGM-2 were added and allowed to bind for 1 h, after which any unbound cells were removed and fresh EGM-2 added. The Bioptechs FC2 closed chamber system with both chamber and objective temperature control was then mounted onto a Zeiss LSM510 based around an AxioVert 100 M. An argon 25 mW (Coherent) laser was employed for GFP excitation (458 nm excitation, 505 nm long-pass filter, laser power 3% transmittance) and simultaneous brightfield phase microscopy. Employing a × 63 Plan-Apochromat 1.4 NA oil/phase objective lens, images were captured at a resolution of 512 × 512 pixels with a pixel dwell time of 1.76 *μ*s. The detector pinhole was set to one airy unit with a stage motor resolution of 1 *μ*M. Visualisation of the data sets was carried out from coverslip to top of the BMEC layer every 30 min over 10 h.

Scoring of data was performed using the Zeiss LSM Image browser viewing time *vs* three-dimensional (3D) axis. A GFP-positive cell was scored as to its position in relation to the BMEC cell layer (Table 1). A ‘+’ score was recorded if a cell made contact with the glass coverslip.

### Cellular invasion assay

Migration of seeded epithelial cells across Matrigel and endothelial cell barriers was measured objectively in invasion chambers. Cell culture inserts (8 *μ*m pore size), coated with Matrigel diluted 1 : 25 with Dulbecco's modified Eagle's media (DMEM), were placed in a 24-well plate containing 1 ml of DMEM/0.1% bovine serum albumin (BSA) with either tissue culture plastic (TCP), BMS or BMEC at the base. PC-3, PNT2-C2, CaP or BPH epithelial cells (1 × 10^5^ cells in 0.25 ml of DMEM/0.1% BSA) were seeded on to the top of the inserts. T140 (10 *μ*M made up in distilled water) was added to the media containing PC-3 cells 30 min prior to plating in the assay. The cells were then incubated at 37°C for 18 h. The inserts were removed, washed in PBS and the noninvading cells together with the Matrigel removed from the insert by wiping with a cotton bud. Inserts were then fixed and stained in 2% crystal violet/20% methanol and air-dried. Cells on the bottom of the insert were counted according to the manufacturer's instructions. Each experiment was carried out in duplicate.

An endothelial barrier was formed by confluent BMEC cells cultured on top of Matrigel within the insert. For this, assay inserts were fixed in methanol/acetone (1 : 1) prior to staining with mouse anti-human pan cytokeratin (1 : 200) followed by biotinylated rabbit anti-mouse secondary antibody (1 : 400). This was then visualised by incubation with streptavidin–HRP complex and DAB substrate prior to counter staining with haematoxylin.

### Immunohistochemistry

Prostate chips were obtained from consenting male subjects undergoing surgery for bladder outflow obstruction from malignant (CaP) or nonmalignant BPH. The tissue was fixed in formalin, paraffin embedded and sectioned. Prostatic bone marrow metastases from 8 mm trephine core iliac crest biopsies taken with informed consent from men undergoing subcapsular orchidectomy for untreated CaP were sectioned and undecalcified. The paraffin-embedded sections were first dewaxed followed by citrate antigen retrieval. Samples were stained with IgG, anti-pan cytokeratin (1 : 200) and CXCR4 (5 *μ*g ml^−1^) according to protocols given above.

### Flow cytometry/CXCR4 analysis

The cell lines PC-3, PNT2-C2 and epithelial cells from patients with CaP or BPH (cultured as described above and used at passage 1) were fixed with 4% formaldehyde/PBS and labelled with CXCR4 PE conjugate in PBS. Cells were analysed using a FACScan flow cytometer (Becton Dickinson). PE was excited at 488 nm and the emission was detected at 565±15 nm band pass. At least 50 000 events were analysed to achieve a significant population for analysis. Analysis of results was performed using WinMDI 2.8.

## RESULTS

### Measurement of PC3-GFP invasion through BMEC

**Figure 1:**
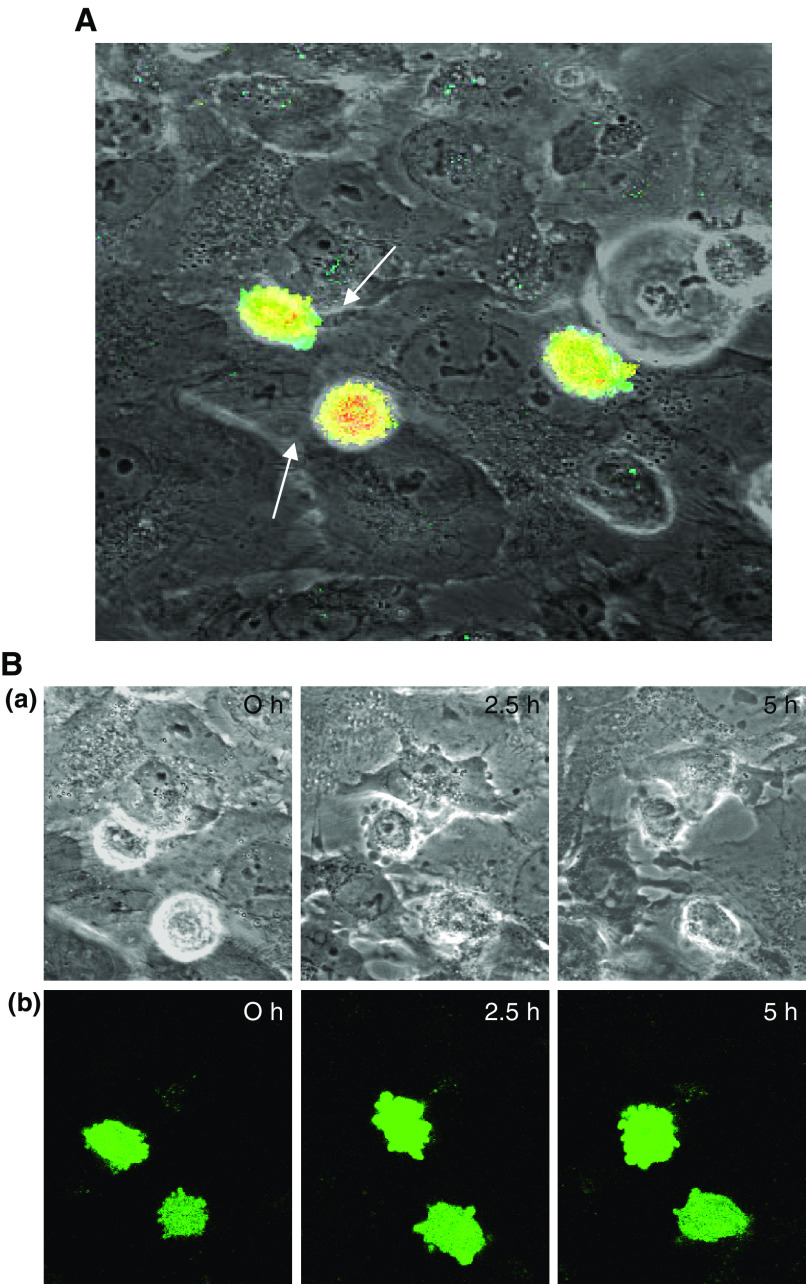
(**A**) PC3-GFP cells after 60 min in culture with BMEC cells. Arrows indicate the joints between endothelial cells. (**B**) (a) Confocal time lapse of PC3-GFP cells interacting with BMEC monolayer showing endothelial cell retraction (arrowed) over a 5 h time frame. (b) Corresponding fluorescent confocal time lapse of PC3 GFP cells.

Previously, Scott *et al* (2001) found that CaP cells bind to BMS and bone marrow endothelial primary cells (BME) in preference to TCP, human umbilical vein endothelial cell line (HUVEC) and prostate fibroblasts. To examine this phenomenon more closely, with particular reference to binding and invasion, we used the GFP-transfected PC-3 cell line in conjunction with BMEC using confocal microscopy. We found that most of the PC3-GFP cells bound within 60 min and further to Scott *et al* (2001), we found that they had a marked tendency to bind at endothelial junctional regions (86.26±7.12%; *P*=0.003). Figure 1A shows three PC3-GFP cells at their binding sites after 60 min in contact with the BMEC. Each endothelial cell can be distinguished clearly and the arrows indicate the junctions at areas where each of the PC3-GFP cells were bound.

These cells were studied using time-lapse confocal microscopy over a 10 h period taking a series of Z slices through the PC3-GFP and BMEC layer every 30 min to obtain a 3D image. Figure 1B shows BMEC retraction postbinding of the PC3-GFP cell. At 2.5 h postepithelial binding, there is significant endothelium retraction (indicated by arrows) along two sides of the invading epithelial cell with almost complete retraction of the surrounding endothelial cells after 5 h. During this period, the epithelial cell remains in contact with one of the endothelial cells.

**Figure 2:**
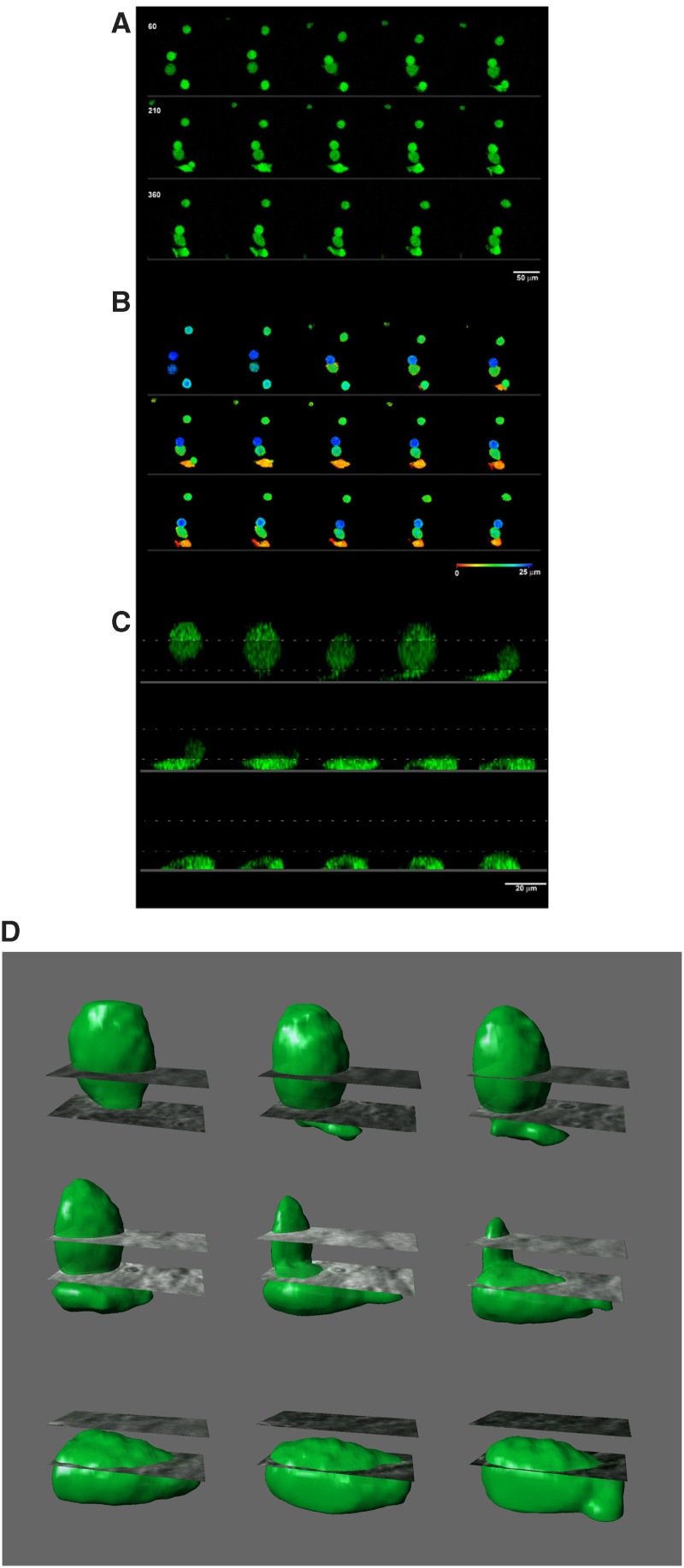
(**A**) Confocal time-lapse of PC3-GFP cells interacting with the BMEC cell layer over 30 min increments (unprocessed volume). (**B**) Height coded 3D image showing the height each cell is positioned at each time point (red indicates the bottom of the endothelial layer, blue the top). (**C**) 3D axial projection of the bottom most cell in the previous images, the dashed line indicates the volume taken by the endothelial layer but does not account for endothelial junctions. (**D**) Four-dimensional isosurface reconstruction of this cell showing changes in cell volume during invasion.

Figure 2A shows the raw unprocessed image of PC3-GFP cells over a period of 7.5 h showing one Z-plane image. For an initial evaluation of the data, a height coded 3D image was generated at each time point via the LSM510 software. Depending on the location in the axial dimension, pixels were pseudocoloured to generate a two-dimensional image map. By analysing multiple Z-plane images for each time point, the GFP signal could be tracked and shown as depth (Figure 2B). Blue indicates 25 *μ*M (top of endothelial layer) down to red 0 *μ*M (base of endothelial layer/coverslip). Over time, the interactions of the PC3-GFP cells with the endothelial layer can be tracked. The bottom cell in this image can be seen to move from pale blue through to green, at which point a small pseudopodial process can be seen to extend downwards to the coverslip (red) and extend outwards along the bottom while the rest of cell remains green. At 210 min, this body is drawn down (red colour) below the BMEC layer. Figure 2C shows this cell as a 3D axial projection. The dashed area represents a visualisation of the volume taken by the endothelial cells (this excludes representation of endothelial joints). Figure 2D shows this cell after further processing. Image analysis was carried out by applying a median filter 3 × 3 × 3 to remove noise. The data were then imported into Imaris (Bitplane AG) where an isosurface was created, a process of 3D thresholding to remove 5% of the pixel values consequently removing background features. The cell of interest was then examined for movement by using the phase data as a reference in the lateral and axial dimension. This advancement in technology has allowed a better understanding of the physical process of invasion of the epithelial cell through the BMEC layer, showing the changes of cell shape.

Data were collected from seven experiments totalling 38 cells, which were scored by two independent investigators regarding the position of each cell over time. Table 1 shows the scoring regime that was adopted to identify the level of invasion through the endothelial layer in stages. We found that 80% of the PC3-GFP cells had bound within 60 min and 100% of the cells had bound to the endothelial layer within 90 min. Within 136 min, 65% of these cells had penetrated to a position half way between the upper and lower surface of the endothelium, while 68% had contact with the coverslip by 170 min. At this point, many of the cells remained static and did not proceed further. However, 29% of the total population did achieve complete invasion through the layer after 232±43 min.

### Stimuli of prostate epithelial cells invasion

Previously, we have shown that the PC-3 cells could be stimulated to invade through the synthetic basement membrane, Matrigel, in response to stimuli from indirect coculture with both primary BME and BMS cells. Therefore, we determined the potential of BMECs and BMS to stimulate PC-3 invasion and determined the utility of this assay to evaluate the invasive responses of primary prostate epithelial cells to different stimuli.

**Figure 3:**
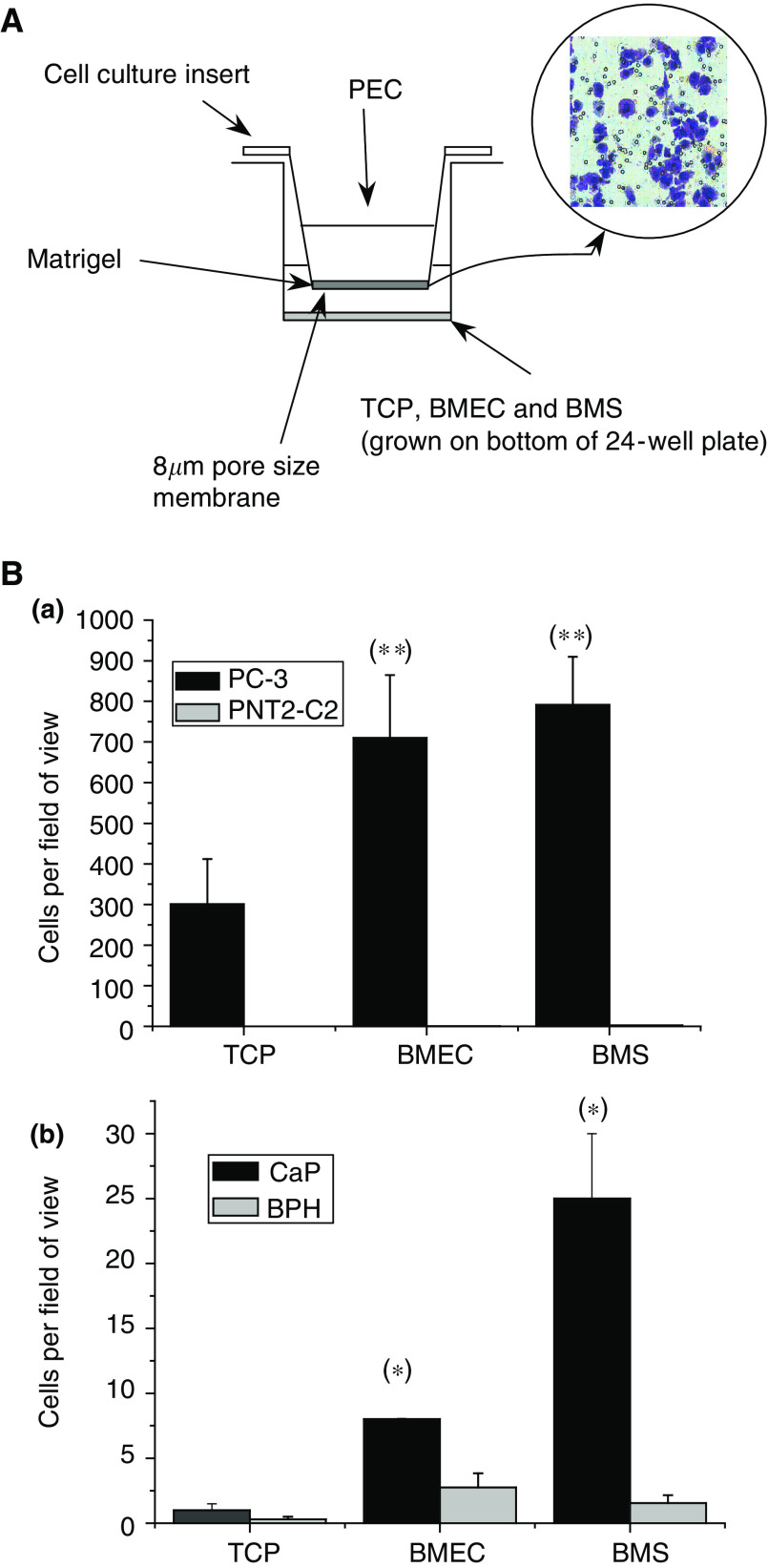
(**A**) Matrigel invasion chamber model seeded with 1 × 10^5^ prostate epithelial cells. Prostate epithelial cell were fixed and stained in 2% crystal violet. Typical field of view of stained cells shown. (**B**) (a) Number of PC-3 and PNT2-C2 cells that invaded through Matrigel towards either TCP, BMEC or BMS (*n*=3). (b) Number of primary cultured prostate cells from patients with CaP (*n*=7) or BPH (*n*=8) that invaded through Matrigel towards either TCP, BMEC or BMS. (*) Denotes significant difference; *P*<0.05. (**) Denotes significant difference to TCP stimulated PC-3; *P*<0.05.

The invasion chamber model, shown in Figure 3A, utilises an 8 *μ*M pore invasion chamber precoated with Matrigel suspended above the stimuli of choice. To eliminate any stimulatory effects of different culture medium, all assays were conducted in the presence of DMEM/0.1% BSA, which did not effect cell viability during the course of the assay. The number of cells that invaded through the Matrigel in response to each stimulus is shown in Figure 3B. The normal prostate epithelial cell line PNT2-C2 was not stimulated to invade through Matrigel by BMECs or by BMS. However, both BMECs and BMS induced PC-3 to invade (*P*=0.033 and 0.039, respectively as compared to TCP control) to a similar degree (*P*=0.69).

Replacing the prostate epithelial cell lines with primary prostate epithelial cells showed that both BMECs and BMS induce malignant prostate epithelial cells to invade (*P*=0.0003 and 0.0009, respectively compared to control). Unlike the cell line model, BMS had a three-fold greater stimulatory effect on malignant primary prostate epithelial cells than the BMECs (*P*=0.0093). Both BMECs and BMS did not significantly induce primary epithelial cells isolated from benign prostates to invade through Matrigel (*P*=0.07 and 0.221, respectively).

### Prostate epithelial invasion in the modelled bone marrow environment

**Figure 4:**
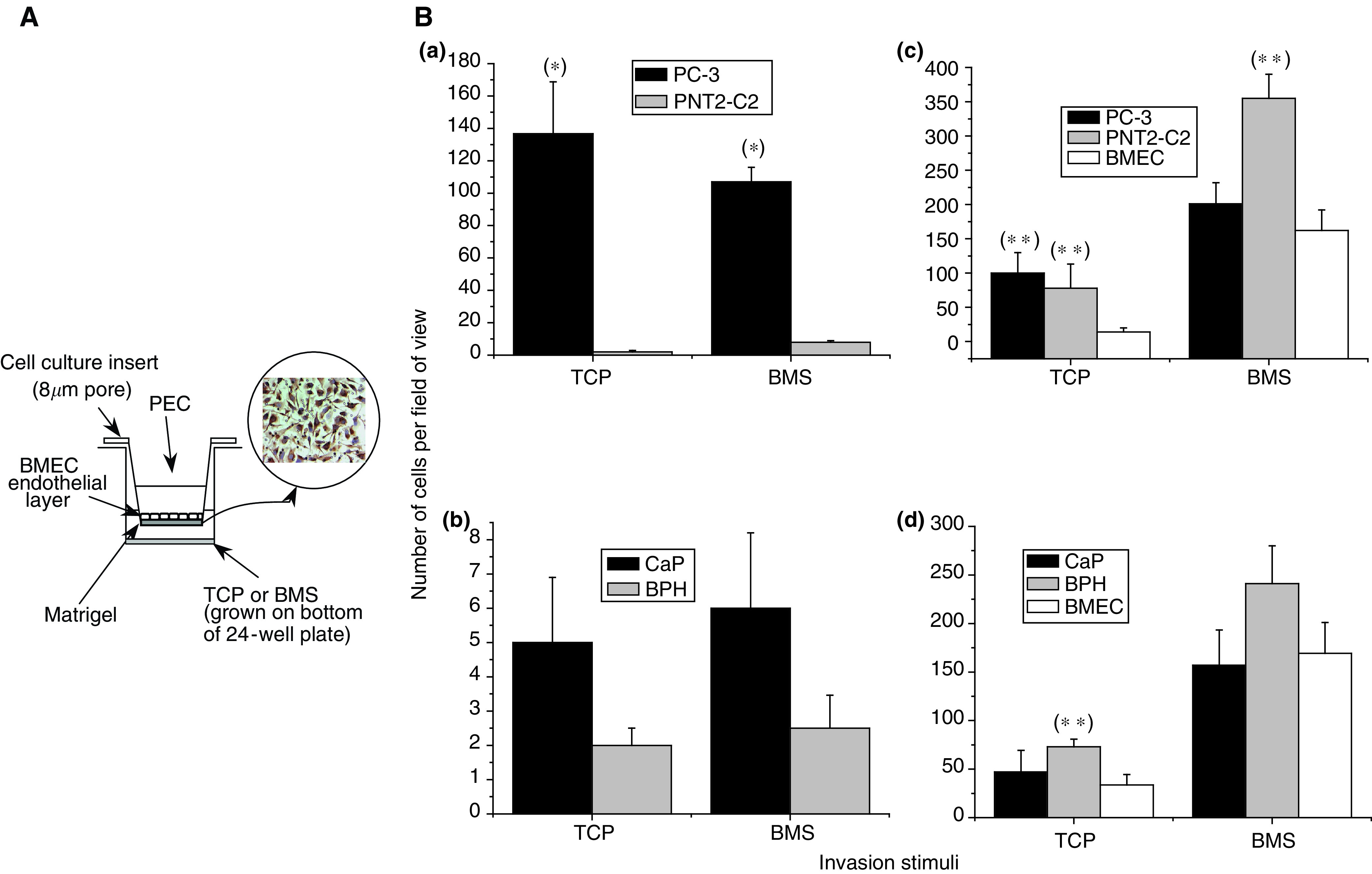
(**A**) Bone marrow endothelium invasion chamber model seeded with 1 × 10^5^ prostate epithelial cells. Prostate epithelial cells fixed in methanol/acetone, stained for cytokeratin by immunohistochemistry and counterstained with haematoxylin. Typical field of view of stained cells shown. (**B**) Number of (a) PC-3 (*n*=5), PNT2-C2 (*n*=3), (b) CaP (*n*=6) and BPH (*n*=5) epithelial cells that invaded through Matrigel and an endothelial barrier towards either TCP or BMS. (*) Denotes significant difference *P*<0.05. The number of BMEC cells that invaded through the Matrigel either on their own or in the presence of prostate epithelial cell lines (c) or primary prostate epithelial cells (d) in response to TCP or BMS. (**) Denotes significant difference to the no epithelial control, *P*<0.05.

To increase the complexity of the invasion model to mirror the *in vivo* bone marrow microenvironment more closely, cell culture inserts (8 *μ*m) were coated with Matrigel and BMEC cells were grown as a monolayer over the top of the formed Matrigel basement membrane. These inserts were placed in a well of a 24-well plate containing either TCP or BMS (Figure 4A). As some endothelial cells migrate through to the base of the insert, we had to be sure that we would be able to distinguish between the endothelial and epithelial cells. Instead of standard crystal violet to stain all cells, we selectively stained for epithelial cells with an anti-pan cytokeratin antibody and counterstained with haematoxylin.

The presence of an endothelial barrier does not prevent invasion of PC-3 cells towards BMS (Figure 4B(a)); however, there is a marked increase in the number of PC-3 cell invading towards TCP with significantly similar numbers invading towards both TCP and BMS stimuli (136±32 and 107±9; *P*=0.498). This is also observed with the malignant primary prostate epithelial cells; however, the numbers of invasive cells were low (5±1.9 and 6±2.2 towards TCP and BMS, respectively; *P*=0.802). Addition of prostate epithelial cells induced migration of BMECs through Matrigel in the presence of TCP (100±30 for PC-3 and 78±35 for PNT2-C2; *P*=0.0117 and 0.065, respectively). The addition of a BMS stimulus induced an overall increase in BMEC invasion with or without prostate epithelial cells. Only PNT2-C2 induced significantly more BMEC invasion than the no prostate epithelial cell control (355±35 *vs* 162±30; *P*=0.00751). Primary prostate epithelial cells had a weaker effect on the BMECs, inducing fewer endothelial cells to invade towards TCP, with only BPH cells stimulating significantly more endothelial cells to invade (73±8 *vs* 34±11; *P*=0.037) than the no prostate epithelial cells control. Unlike the prostate epithelial cell line response, both benign and malignant primary prostate epithelial cells did not induce significantly more endothelial invasion in the presence of a BMS stimulus than the no prostate epithelial cells control (*P*=0.8233 and 0.2208 for CaP and BPH, respectively).

### Inhibition of CXCR4 signalling by T140

The utility of these invasion models to analyse the role of specific stimulators and inhibitors of prostate epithelial cells was assessed. It has been shown that SDF-1, expressed by both BMS and BMECs (Aiuti *et al*, 1997), and its receptor CXCR4 play an important role in targeting not just blood cells but also prostate epithelial cells towards the BMS (Taichman *et al*, 2002; Sun *et al*, 2003). Therefore, we examined the ability of SDF-1, and its specific peptide inhibitor T140, to stimulate invasion of prostate epithelial cells in our models.

**Figure 5:**
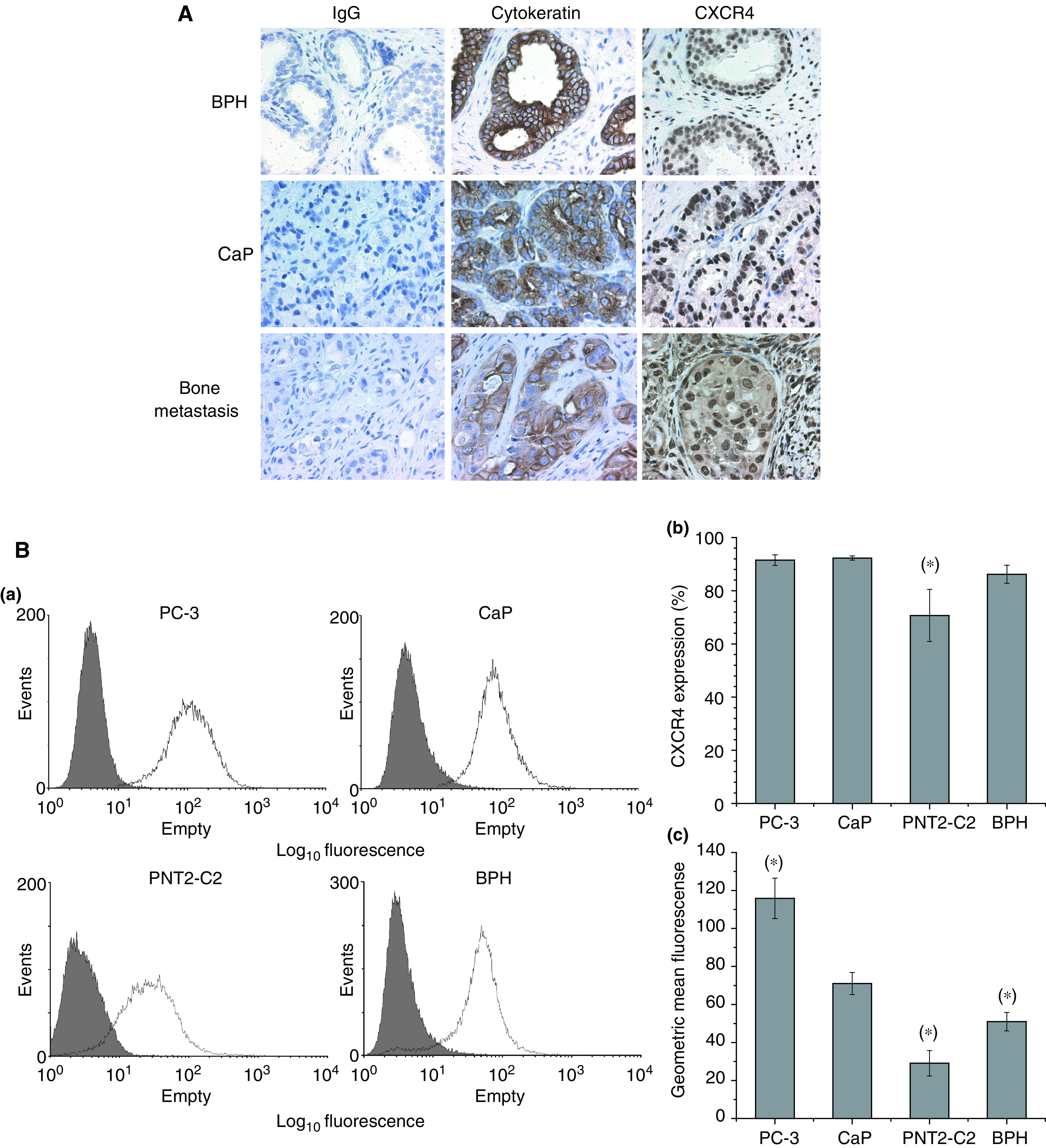
(**A**) Immunohistochemical staining of prostate sections taken from patients with BPH, CaP or bone metastases. These sections were labelled with IgG1, anti-pan-cytokeratin and anti CXCR4 (clone 12G5) and stained with DAB (brown). These sections were counterstained using haematoxylin (blue). (**B**) FACs CXCR4 receptor expression in primary cultured prostate from patients with CaP (*n*=6) or BPH (*n*=5) and on the PC-3 and PNT2-C2 prostate cell lines (*n*=3). (a) Histograms showing expression of CXCR4 by each cell culture as compared to IgG control (shaded histogram). (b) Percentage of cells expressing the CXCR4 receptor; (*) denotes significant difference to CXCR4 expression in patients with CaP; *P*<0.05. (c) Level of expression of CXCR4 by each prostate epithelial population; (*) denotes significant difference to CaP, *P*<0.05.

Immunohistochemical analysis of prostate sections taken from patients with benign disease, localised CaP or bone metastases (Figure 5A) showed that both BPH and localised CaP expressed high levels of CXCR4 within the nucleus. Prostate bone metastases, by contrast, express high levels of CXCR4 in both the nucleus and the cytoplasm. FACs analysis of PC-3 and PNT2-C2 cell lines and primary benign and malignant prostate epithelial cells (Figure 5B(b)) shows that similar percentages of PC-3, BPH and CaP epithelial cells express CXCR4 (*P*>0.05) but expression was lower in PNT2-C2 cells (23% reduction; *P*=0.0048 as compared to CaP). However, there was considerable and significant variation (*P*<0.05) in the levels of CXCR4 expression between primary and cell lines and in relation to their type. PC-3 cells expressed the highest levels of CXCR4 followed by primary CaP and BPH prostate epithelial cells (geometric mean fluorescence of 115.8±10.7, 71±5.8 and 51±4.8, respectively). The lowest level of expression was seen in the transformed normal prostate epithelial cell line PNT2-C2 with a geometric mean fluorescence of 29±6.7.

**Figure 6:**
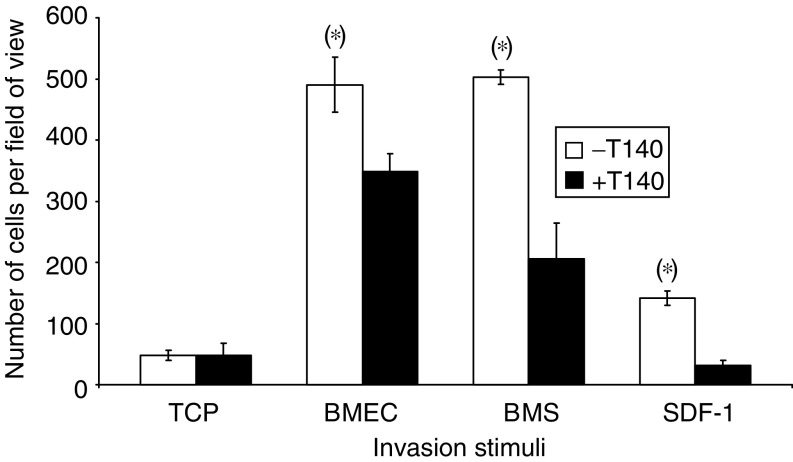
PC-3 invasion through Matrigel towards TCP, BMEC, BMS and SDF-1 (15 ng ml^−1^) with or without the CXCR4 receptor blocked with the CXCR4-specific T140 (*n*=3). (*) Denotes significant difference *P*<0.05.

The effect of the specific CXCR4 inhibitor, T140 (Tamamura *et al*, 2001, 2003), on prostate epithelial cell invasion was then assessed in the Matrigel invasion model with the PC-3 cell line (Figure 6). The invasive stimulatory effect of the maximal invasive dose of SDF-1, 15 ng ml^−1^ (titration data not shown), was compared to the invasive stimuli from BMECs and primary BMS with or without 10 *μ*g ml^−1^ T140 (concentration required to inhibit completely SDF-1 signalling in prostate epithelial cells (titration data not shown)). Bone marrow endothelial cell lines and BMS both induced PC-3 invasion across the Matrigel barrier, as did 15 ng ml^−1^ SDF-1, although SDF-1 induced significantly less PC-3 invasion than either the BMECs (141±12 *vs* 490±36 *P*=0.0007) or the BMS (141±12 *vs* 503±29 *P*=0.0004). Addition of 10 *μ*M T140 resulted in a complete block in invasion towards SDF-1 (*P*=0.0103). However, unlike SDF-1-induced invasion, T140 only reduced the levels of PC-3 invasion incompletely (29% towards BMEC (*P*=0.011) and by 59% towards BMS (*P*=0.001)).

## DISCUSSION

We have previously developed models allowing the study of the interactions between malignant prostate epithelial cells and endothelial or BMS layers (Lang *et al*, 1997, 1998; Scott *et al*, 2001). We have developed these models to target the blood/BMS endothelial barrier specifically, thereby allowing the visualisation not just of the sites of binding but also of the prostate epithelial invasive process. The development of such models enables the characterisation and comprehension of the mechanisms of metastasis in prostate and other cancers to be carried to a greater depth. Understanding and modelling this process will not only elucidate new therapeutic targets but also may help to elaborate their mode of action. In addition, it will help to explore the potential therapeutic benefit of novel chemotherapeutic agents such as T140 (Tamamura *et al*, 2001).

We have shown that prostate epithelial cells bind preferentially to primary human bone marrow endothelial cells and that only malignant prostate epithelial cells can invade through a matrigel basement membrane (Scott *et al*, 2001). With a view to creating robust models of epithelial/endothelial interactions, we have replaced the primary BME cells with the BMEC cell line. This line displays characteristics indistinguishable from human primary BME cultures (Almeida-Porada and Ascensao, 1996). We have also utilised an GFP-expressing invasive prostate epithelial cell line, PC3-GFP (Sharrard and Maitland, 2000), to allow visualisation and measurement of the process of cellular trans-migration by confocal microscopy.

Our results show that the prostate epithelial cells bind rapidly to the BMEC cell layer. This process is complete within 90 min, confirming the findings in previous clinical studies whereby prostate cells released into the circulation during surgery were removed from the peripheral blood within 2 h (McIntyre *et al*, 2002). Our results show that the location of the binding is very specific, with all the PC3-GFP cells binding at endothelial cell junctions. It has been shown that prostate epithelial cells interact directly with the BME cells, initially via selectins and this interaction is then stabilised by integrin binding (Orr *et al*, 2000). These are not the only binding steps, since antibodies to CD11a, CD18, LFA-1 and CD31 have also been shown to interfere with the binding process (Lehr and Pienta, 1998).

Once bound, prostate epithelial cells induce endothelial cell retraction. The precise mechanism of this process, which is an essential component of cellular trans-migration, is at present unclear. A major component of the signalling cascade modulating endothelial permeability is the intracellular level of Ca^2+^ (Curry, 1992). Studies by Lewalle *et al* (1998) demonstrated that the binding of breast epithelial cells to HUVECs induced a transitory rise in HUVEC intracellular concentration of Ca^2+^ resulting in endothelial retraction and epithelial migration. This rise in Ca^2+^ levels and retraction of the endothelial layer is entirely dependent on cell-to-cell contact and inhibiting this rise in intracellular Ca^2+^ concentration inhibited breast epithelial trans-endothelial migration. The binding of prostate epithelial cells and melanoma cells also have induced raised intracellular Ca^2+^ levels (Pili *et al*, 1993), which correlated with increased binding of the epithelial cells. Previous studies by our group (Montague *et al*, 2004) have shown that treatment of BMEC with the bisphosphonate zoledronic acid, a potent calcium chelating agent and blocker of the mevalonate transduction pathway, tightens the endothelial-to-endothelial cell binding in the absence of prostate epithelial cells. It is also a potent inhibitor of prostate epithelial trans-endothelial migration. However, the tightening of the endothelial-to-endothelial cell bonding is not observed with high doses (100 *μ*M) of the weaker bisphosphonate pamidronate, or with EDTA, both of which are potent Ca^2+^ chelating agents. Therefore, it is unlikely that endothelial retraction relates to decreased levels of extracellular Ca^2+^, although the effects on intracellular Ca^2+^ levels at the higher concentrations observed in relation to endothelial binding experiments are unknown (Montague *et al*, 2004).

The effect of agents such as zoledronic acid in reducing the ability of PC-3 cells to invade across endothelial barriers towards BMS suggest that a major component affecting migration is inhibition of transduction pathways related to the Rho axis. Zoledronic acid is an effective inhibitor of the mevalonate pathway, which is known to be related to the Rho pathway through Ras linkage (Virtanen *et al*, 2002). Inhibition of this pathway affects downstream prenylation of small GTPases such as Rho, which is known to be involved integrally in cell motility. Therefore, an early event following integrin *β*1-binding may be the induction of a specific pathway or pathways, which relate to Rho. Whether or not this is a consequence of flux in intracellular calcium levels within endothelial cells remains to be determined. The role of integin *β*1 and the interaction between the prostate epithelial cell and the endothelial tight cell junction in relation to induced rises in intracellular Ca^2+^ concentrations is certainly worthy of further study.

Malignant prostate cells migrate across the endothelial barrier in a manner similar to melanoma cells (Voura *et al*, 1998). The prostate epithelial cells bind rapidly to the endothelial junctions, where they begin to penetrate the endothelial barrier. The prostate epithelial cells show marked membrane blebbing and lamellipodial formation on the lower surface of the cell (Figure 1) at the point of contact between the two cells. The prostate epithelial cell then generates a pseudopodial extension, which penetrates the endothelial cell layer, the endothelial cells retract and the prostate cell moves through the endothelial barrier. As with the migration across endothelia observed in melanoma cells, prostate epithelial trans-migration is considerably slower than leukocyte trans-migration, with 29% of cells completing the transit within 4 h. This extravasation time is comparable to that observed for melanoma cells (Voura *et al*, 1998), rat ascites hepatoma cells (Ohigashi *et al*, 1989) and other tumour cells (Kramer and Nicolson, 1979). However, it has been shown that over 50% of monocytes can cross an endothelium within the first hour of contact (Sandig *et al*, 1997) without inducing endothelial cell retraction. This difference may be due to the fact that epithelial cells are larger than monocytes and therefore require retraction of the endothelium, thereby resulting in the significantly increased time of invasion.

Figure 4B shows that the addition of a BMEC barrier does not inhibit PC-3 invasion but appears to act as a stimulus in the TCP control. As shown in Figure 3, both BMS and BMECs are powerful invasion stimuli, possibly inducing maximal invasion within our assay system. This would explain why we do not observe an enhanced effect in the combined BMEC BMS assay model. We have also shown that trans-endothelial migration by prostate epithelial cells induces the invasion of bone marrow endothelial cells across the Matrigel basement membrane. The exact nature of this basement membrane invasion by the bone marrow endothelial cells is unknown but may be a result of the weakening of the basement membrane itself due to prostate epithelial proteolytic secretions, a phenomenon known to occur during prostate epithelial migration and metastasis (Hart *et al*, 2002). However, Figure 4B shows that in the presence of both PNT2-C2 and BPH there is significant BMEC invasion without epithelial invasion, which suggests that the overall integrity of the basement membrane has remained intact. Figure 4B also shows a marked increase in endothelial cell invasion in the presence of BMS suggesting that the BMECs may be responding to factors other than the potential breakdown of the basement membrane by the prostate epithelial cell, for example, VEGF or other angiogenic factors.

Taichman *et al* (2002) showed that prostate epithelial cells bind to both osteosarcoma cell lines, MG-63 and SaOS-2, and to human bone marrow endothelial cells. Previously, we have shown that both benign and malignant primary prostate epithelial cells bind preferentially to BMS (Lang *et al*, 1997, 1998) and to BME cells to a similar degree (Scott *et al*, 2001) and that prostate epithelial cells shed intraoperatively into the peripheral blood during trans-urethral resection of the prostate are undetectable within 2 h of the end of the operation (McIntyre *et al*, 2002). Since both benign and malignant prostate epithelial cells express CXCR4 and bind preferentially to BME cells, a process which is enhanced by SDF-1 signalling, it is highly likely that prostate epithelial cells that are released into the circulation, whether benign or malignant, are rapidly removed from the blood by binding to the bone marrow endothelium and possibly to endothelial surfaces in other capillary beds.

The chemokine receptor, CXCR4, and its endogenous ligand SDF-1 have been shown to be key components in both chemokine-induced leucocyte trafficking (Aiuti *et al*, 1997, 1999; Hamada *et al*, 1998) and the migration of malignant epithelial cells to the BMS (Koshiba *et al*, 2000; Muller *et al*, 2001; Robledo *et al*, 2001; Murakami *et al*, 2002; Schrader *et al*, 2002; Taichman *et al*, 2002; Burger *et al*, 2003; Sun *et al*, 2003). This has led to the hypothesis that CXCR4 is the key component of metastatic implantation in bone marrow and that it represents an important therapeutic target for metastatic bone disease. Blockade of the CXCR4 signalling in malignant breast epithelial cells either by neutralising antibodies (Muller *et al*, 2001) or by peptide antagonists such as T140 (Tamamura *et al*, 2003) has been shown to inhibit metastasis *in vivo*.

Utilising our *in vitro* assays of metastasis, we sought to determine the influence of SDF-1 signalling via CXCR4 as a stimulus for invasion toward BMS. The analysis of CXCR4 expression by metastatic and benign cell lines, primary prostate epithelial cells and tissue sections of BPH, primary cancer and bone metastases demonstrate that all prostate epithelial cells express CXCR4, although the levels and localisation of expression vary according to the type of disease affecting the cell. Our results correlate with the observation of Spano *et al* (2004) that CXCR4-positive nuclear staining of non-small-cell lung cancer correlates with a significantly better outcome. Both BPH and localised CaP sections show strong CXCR4 nuclear staining while the prostate bone metastases, a poor prognostic indicator, showed strong CXCR4 nuclear and cytoplasmic staining. Our results also confirm the observation of Sun *et al* (2003) that the level of CXCR4 expression increased with increasing malignancy, with the greatest expression being observed in the aggressively metastatic cell line PC-3 and in the human bone metastasis sections. This increasing expression suggests that CXCR4/SDF-1 signalling may be one of the key signalling pathways for metastatic spread to the bone. The importance of this pathway was demonstrated by Taichman *et al* (2002), utilising a matrigel basement membrane invasion assay to show that SDF-1 signalling induced both DU145 and PC3 cells to invade. However, this study only utilised recombinant SDF-1 and anti-CXCR4 antibody inhibitors and therefore did not determine whether SDF-1/CXCR4 signalling pathway was the sole chemo-attractant in the spread of prostate epithelial cells to the bone. Our study confirms that SDF-1 is a potent stimulus for invasion but the level of that invasion is significantly less than that seen by using either BMEC cells and/or BMS alone. This measured phenomenon is reinforced by the observation that use of a specific CXCR4 antagonist peptide (T140), at a concentration which blocks prostate epithelial cell invasion in response to maximum levels of SDF-1 signalling, does not block invasion towards either BMEC or BMS completely. Thus, it is confirmed that while the CXCR4/SDF-1 signalling pathway is important in prostate epithelial metastasis, it is not the only signalling pathway involved. This study of CXCR4/SDF-1 signalling in CaP metastasis to the bone marrow demonstrates the need for more integrated models of bone metastasis. The BMS is a highly complex environment supporting haematopoiesis and as such produces a wide variety of chemokines, which may attract metastatic epithelial cells. While basic models may identify individual ‘key’ components in metastatic disease, it is necessary to utilise more complex, coculture models to determine the exact nature of each signalling pathway within the complex bone marrow environment.

We have shown that it is possible to generate *in vitro* models that are able to mimic the highly complex bone metastatic environment. These models show that epithelial–endothelial binding occurs rapidly and that trans-endothelial migration is initiated at the intercellular joints between multiple endothelial cells. This results in endothelial cell retraction and epithelial invasion within 4 h. We have also shown that while CXCR4/SDF-1 signalling is an important stimulus for epithelial invasion towards the bone marrow, it is not the only stimulus emanating from the bone marrow attracting metastatic prostate epithelial cells.

The main advantage of such models over the standard mono culture/stimuli variations of Bowden invasion chambers are that they allow the study of proposed components of the metastatic process and the effect of novel chemotherapeutic agents within the relevant background of chemokines and growth factors.

## Figures and Tables

**Table 1:** Time taken (min) for PC3-GFP cells to invade through the BMEC layer and the percentage of test cells that attained this

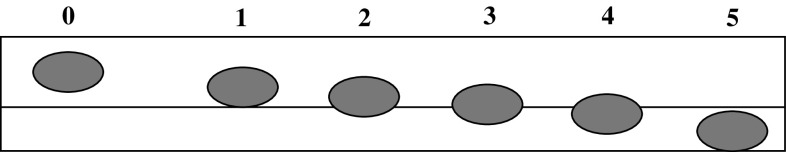				
	**Time (min)**			
	**Stage 1**	**Stage 2**	+	**Stage 5**
Mean (*n*=38)	90	136	170	232
Std error (±)	13	17	20	43

% Cells	100	65	68	29

Key: PC3-GFP cell volume penetrating upper surface of BMECs: 0=unbound; 1=bound; 2=25% volume invaded; 3=50% volume invaded; 4=75% volume invaded; 5=100% total invasion; +=any part of the cell makes contact with the coverslip.
